# A phylogenomic analysis of *Escherichia coli* / *Shigella* group: implications of genomic features associated with pathogenicity and ecological adaptation

**DOI:** 10.1186/1471-2148-12-174

**Published:** 2012-09-07

**Authors:** Yan Zhang, Kui Lin

**Affiliations:** 1 College of Life Sciences, Beijing Normal University, No 19 Xinjiekouwai Street, Beijing, 100875, China

**Keywords:** Phylogenomics, Collinearity, Pathogenicity, *Escherichia coli*, Adaptation

## Abstract

**Background:**

The *Escherichia coli* species contains a variety of commensal and pathogenic strains, and its intraspecific diversity is extraordinarily high. With the availability of an increasing number of *E. coli* strain genomes, a more comprehensive concept of their evolutionary history and ecological adaptation can be developed using phylogenomic analyses. In this study, we constructed two types of whole-genome phylogenies based on 34 *E. coli* strains using collinear genomic segments. The first phylogeny was based on the concatenated collinear regions shared by all of the studied genomes, and the second phylogeny was based on the variable collinear regions that are absent from at least one genome. Intuitively, the first phylogeny is likely to reveal the lineal evolutionary history among these strains (i.e., an evolutionary phylogeny), whereas the latter phylogeny is likely to reflect the whole-genome similarities of extant strains (i.e., a similarity phylogeny).

**Results:**

Within the evolutionary phylogeny, the strains were clustered in accordance with known phylogenetic groups and phenotypes. When comparing evolutionary and similarity phylogenies, a concept emerges that *Shigella* may have originated from at least three distinct ancestors and evolved into a single clade. By scrutinizing the properties that are shared amongst *Shigella* strains but missing in other *E. coli* genomes, we found that the common regions of the *Shigella* genomes were mainly influenced by mobile genetic elements, implying that they may have experienced convergent evolution via horizontal gene transfer. Based on an inspection of certain key branches of interest, we identified several collinear regions that may be associated with the pathogenicity of specific strains. Moreover, by examining the annotated genes within these regions, further detailed evidence associated with pathogenicity was revealed.

**Conclusions:**

Collinear regions are reliable genomic features used for phylogenomic analysis among closely related genomes while linking the genomic diversity with phenotypic differences in a meaningful way. The pathogenicity of a strain may be associated with both the arrival of virulence factors and the modification of genomes via mutations. Such phylogenomic studies that compare collinear regions of whole genomes will help to better understand the evolution and adaptation of closely related microbes and *E. coli* in particular.

## Background

*Escherichia coli* is one of the most important model organisms in both biology and medicine. Many major findings have emerged from the study of *E. coli*, including bacterial conjugation, recombination and genetic regulation. More importantly, *E. coli* plays important roles in the intestinal tract of humans and other vertebrates, especially in the lower section. There are more than a billion *E. coli* cells in the intestines of a healthy human [1]. Unfortunately, several *E. coli* strains can cause intestinal and extraintestinal diseases, such as diarrhea, urinary tract infection, septicemia, pneumonia and meningitis, in humans and animals [2]. The availability of an increasing number of complete *E. coli* genomes has revealed that *E. coli* exhibits high diversity at the whole-genome level. Comparative genomic analyses have demonstrated that the diversity among natural isolates of *E. coli* is extraordinarily high, and the average genome-wide conservation across different strains is less than 50% [1]. Therefore, *E. coli* is an ideal candidate for studying how the relationship between a bacterium and its host can fluctuate between commensalism and pathogenicity [3].

In general, at the whole-genome level, two main categories of methods are used to assess phylogenetic relationships among prokaryotes (i.e., phylogenomic analysis). One method is based on the concept of orthology, in which sequence alignment is the core computational method. Many approaches, such as gene content, gene order, multilocus sequence typing (MLST) and super-tree or super-matrix methods, belong to this category [1,4-6]. Another approach is based on the frequencies of *K*-mer oligonucleotides and does not employ an alignment [6,7]; this type of method emphasizes the importance of genome content and organization. Intuitively, for phylogenomic analysis, we are seeking one or a set of genomic features that can be used as indicators/markers to robustly and correctly reveal the evolutionary relationships among a group of organisms of interest. In addition, we are also interested in features that are functional units, which could act as a bridge between genomic diversity and phenotypic differences. Within bacterial systems, the concept of an operon satisfies these two criteria. Operons are groups of genes that exhibit physical clustering within the genome and are typically transcribed in a single mRNA [8]. Genes within the same operon usually have related functions, and some of these genes may be employed in the same pathway. Regulatory genes are also commonly located in close proximity to the genes that are being regulated [8]. Although certain operons may comprise genes with no clear functional relationship, these genes may be required under the same environmental conditions even though they are involved in different pathways [9]. Unfortunately, many, if not all, operons predicted in databases to date consist only of structural genes that lack expressional regulatory elements. It is well known that the correct expression of genes must remain faithful to the specific genetic background. In addition, certain relatively large clusters of genes that have related functions, but do not belong to the same operon, have been described [10]. Therefore, it is currently assumed that predicted operons may be difficult to use in practice as indicators/markers for phylogenomic studies. With the availability of an increasing number of closely related or intraspecific prokaryotic genomes, as well as the advent of whole-genome alignment algorithms [11,12], there is an opportunity to implement phylogenomic analyses of the evolution and ecological adaptation of these organisms on the whole-genome scale. To this end, we chose one type of genomic feature, called locally collinear blocks (LCBs), to study the evolutionary relationships and potential ecological adaptations of *E. coli* on the whole-genome scale. In principle, LCBs from closely related organisms or within one species should contain useful phylogenomic signals regarding their evolutionary histories. Each LCB, also known as a collinear region, is a region of DNA sequence that is shared by two or more genomes that are being studied [11]. Clearly, if an LCB is sufficiently large, it is likely to contain one or more consecutive genes with related functions in addition to their regulatory regions. Therefore, LCBs that are present or absent in either genome may satisfy both of the aforementioned criteria for feasible genomic markers; if these criteria are met, the analysis of LCBs should reveal a comprehensive history of the evolutionary and ecological adaptation of *E. coli* genomes.

To test our hypothesis, we studied the vertical and phenetic relationships of 34 strains of *E. coli* at the level of LCBs. First, we identified potential LCBs using the Mugsy program [12]. Next, we divided the LCBs into two groups according to their occurrence among the strains: core and variable LCBs. The core LCBs are the set of collinear regions shared by all of the studied strains, whereas the variable LCBs are the set of collinear regions that were absent in at least one of the 36 strains. Then we constructed two phylogenies based on the LCBs from each of these two groups. The phylogeny based on core LCBs tends to reflect the vertical evolutionary history of the strains (i.e., the evolutionary phylogeny). In contrast, the second phylogeny, based on the variable LCBs is likely to reveal the whole-genome similarities of extant strains (i.e., the similarity phylogeny). In the evolutionary phylogeny, the strains were clustered into groups as known phylogroups. Within each phylogroup, strains were grouped according to their respective pathotypes. These patterns indicate that it is feasible to use LCBs as indicators/markers to infer intraspecific phylogenies. We also found that the B2 phylogroup occured at the base of the evolutionary phylogeny, thereby suggesting that the ancestor of *E. coli* / *Shigella* was an opportunistic pathogen. Such a pathogen may be harmless under certain environmental conditions and pathogenic in other settings [7]. A comparison of the evolutionary and similarity phylogenies shows that *Shigella* may have at least three origins. We scrutinized the common properties of *Shigella* that were missing in other *E. coli* genomes and found that the common LCBs from their genomes were mainly influenced by mobile genetic elements. This finding implies that *Shigella* may have experienced a convergent evolution event via horizontal gene transfer (HGT) and acquired similar phenotypes during the course of evolution. Interestingly, by inspecting specific branches of the similarity phylogeny and correlating the branch support of LCBs with key branches in the evolutionary phylogeny, we identified putative LCBs that may be relevant to the pathogenicity of certain strains. Moreover, by analyzing the annotated genes within these regions, additional details on the evidence associated with pathogenicity were revealed, which may provide clues for further experimental evaluation. We believe that such phylogenomic studies, which examine collinear regions of whole genomes, will help to better understand the evolution and adaptation of microbes and *E. coli* in particular.

## Results

### Identification of the LCBs for phylogenomic analysis

**Figure 1:**
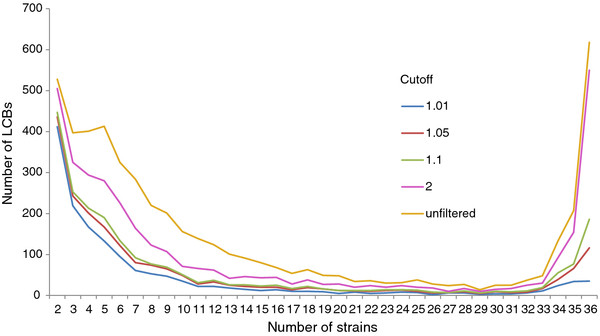
**The distribution of the number of LCBs shared by the strains.** Before being filtered, 528 and 618 LCBs were shared by 2 and 36 strains, respectively (orange). After being filtered using a cutoff of 1.01, 412 and 35 LCBs shared by 2 and 36 strains, respectively, remained (blue). A cutoff value of 1.01 means that the length of the gaps in the LCBs is less than 1% of the length of the non-gap regions.

Among closely related genomes, phylogenetic information should be inferred at the whole-genome scale. To precisely reconstruct the evolutionary phylogeny of the strains studied here, we identified as many potential collinear genomic regions as possible using a tool named Mugsy. Of the identified LCBs, we observed many gaps within several collinear regions. To ensure the quality of the LCB alignment, we filtered the collinear regions using the cutoff values defined in the *Methods*. A smaller cutoff value corresponded to fewer allowed alignment gaps. The occurrence of LCBs was not uniform among the strains, and their distribution displayed a U-like shape. The identified collinear regions tended to be either shared by most (right portion of Figure 1) or a few strains (left portion of Figure 1). Interestingly, this pattern is identical to that observed for individual genes [1]. This may be due to the fact that the LCBs we identified are mainly composed of genes. At the operon level, it has reported that bacterial genomes usually contain a small number of highly conserved operons and a much larger number of unique or rare ones [13]. After operons form, many of them are lost through the deletion of one or more genes contained within the operon [9]. Therefore, few operons are conserved across all or even the majority of genomes. LCBs existing in only a few strains are more than that present in all or most of the strains. This pattern is similar with that of operons, indicating that the collinear regions identified here might appear to have experienced an evolutionary history similar to that of operons. After being filtered using a cutoff value of 1.01, 412 and 35 LCBs remained, which are shared by 2 and all 36 strains, respectively. These 35 core LCBs with a combined length of 62,605 bp comprise ~1% of the average length of *E. coli* genomes (Table 1).

**Table 1:** The length of the 35 core LCBs

**LCB ID**	**Length (bp)**
41	12,529
261	5,326
350	4,263
366	4,095
428	3,506
435	3,457
474	3,146
527	2,805
539	2,694
629	2,169
640	2,125
678	1,938
692	1,814
798	1,388
810	1,323
879	1,125
896	1,074
918	1,009
964	1,062
974	898
991	852
1047	727
1178	535
1182	533
1219	489
1225	487
1231	480
1781	176
1792	174
2325	101
2524	83
2789	65
2836	62
3282	63
3530	32
**Total**	62,605

### Two types of phylogenies for *E. coli / Shigella*

**Figure 2:**
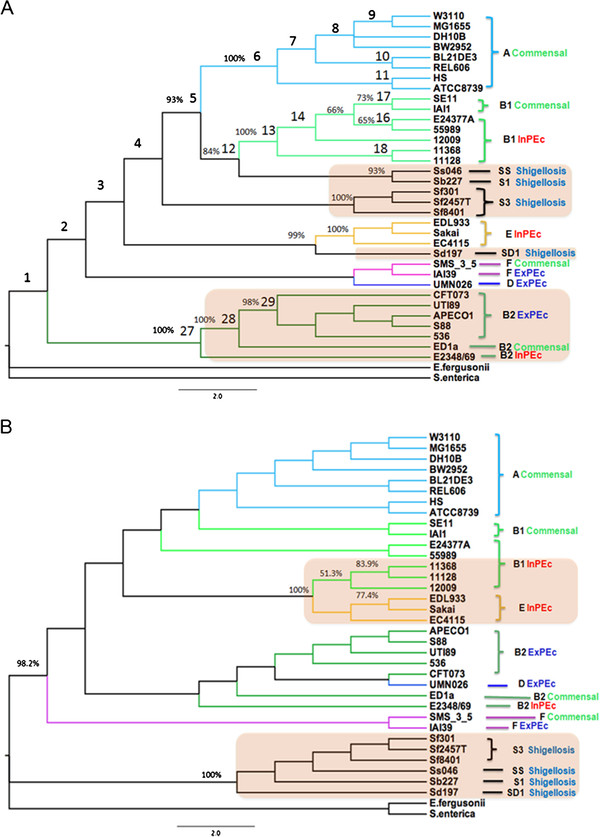
**The evolutionary and similarity whole-genome phylogenies of*E. coli*/*Shigella*.** (**A**) The maximum parsimony tree was constructed using the concatenated LCBs that are shared by all of the 36 sequenced strains. The reliability of the topology was assessed by bootstrapping with 1000 pseudo-replicates. The phylogroup (A, B1, B2, D, E, F, S1, S3, SS or SD1) and pathotype (Commensal, InPEc, ExPEc, or Shigellosis) of each strain is indicated on the right. (**B**) The neighbor-joining phylogeny was constructed using the Jaccard distances. Only the LCBs that were absent in at least one strain and present in at least two strains were analyzed. The reliability of the topology was assessed by re-sampling the collinear regions 1000 times.

After filtering using a cutoff value of 1.01, 35 and 1493 core and variable LCBs remained, respectively. Based on these two sets of LCBs, we constructed two different phylogenies using the LCBs as the phylogenomic signatures. The first phylogeny (Figure 2A), constructed using the core LCBs, is likely to reflect the lineal evolutionary history among the strains (i.e., the evolutionary phylogeny) [7]. Based on the presence and absence of the variable LCBs, the second phylogeny (Figure 2B) is likely to reveal the similarities among the extant strains (i.e., the similarity phylogeny) [7], especially if the strains experienced convergent or divergent evolution. In the evolutionary phylogeny constructed here, the strains were clustered into groups according to their phylogroups, and within each phylogroup, the strains were clustered corresponding to their pathotypes (commensal, ExPEc or InPEc). These results indicate that core LCBs are good markers of intraspecific relationships. In our similarity phylogeny, however, most strains were not grouped with similar phylogroups or pathotypes.

### The evolution of *Shigella*

*Shigella* was once elevated to the genus status based on its ability to cause a specific type of diarrhea. However, from an evolutionary perspective, all *Shigella* strains should be classified as *E. coli*[14]. In our evolutionary phylogeny, *Shigella* strains were divided into three clades: one clustering with B1, a second with E and a third group independent from other phylogroups. This result is in agreement with the tree of Touchon et al. [1]. In our similarity phylogeny, however, *Shigella* formed a monophyletic clade. This is in agreement with those trees that are likely to reveal the genome similarities [4,7,15]. *Shigella* strains scattered across three groups in the evolutionary phylogeny, while they formed a monophyletic clade in the similarity phylogeny. This pattern suggests that there were multiple origins of *Shigella*, which is in accordance with the findings of Rolland et al. [16], Haggerty et al. [17] and Pupo et al. [14]. However, these results are inconsistent with the theory of Escobar-Paramo et al. [18], who suggested that there was a single origin of *Shigella*. Interestingly, we also observed that most of the genes along the collinear regions specific to these six *Shigella* genomes correspond to transposases, insertion sequences or antigens (Additional file 1: Table S1). This observation may suggest that distantly related *Shigella* strains probably acquired a specific set of genes related to their extant phenotype and underwent convergent evolution. In addition, *Shigella* strains also achieved fitness through the inactivation or loss of genes incompatible with the virulence, which could occur by IS mobilization [19].

### Putative genomic signatures related to pathogenicity

There are three types of factors that may be associated with the pathogenicity of *E. coli*. First, pathogenic strains are considered to differ from non-pathogenic strains primarily by the arrival of virulence factors (VFs) through HGT events [20,21]. Nonetheless, the integration, retention and expression of new incoming genes cannot deviate from the specific genetic background [22]. Second, commensal *E. coli* can modify their genomes through deletions, point mutations or other DNA rearrangements to adapt to specific environments and cause disease in a host [20]. Third, certain types of virulence are coincidental byproducts of commensalism [23].

**Figure 3:**
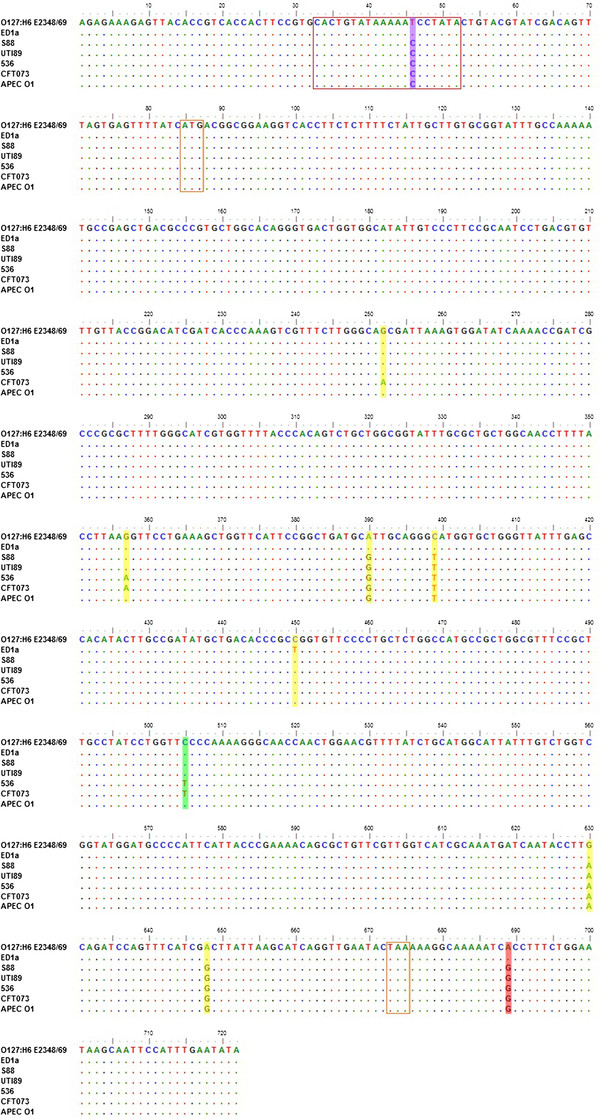
**Substitution variants revealed by the multiple sequence alignment of the seven DNA segments (label 1047).** The red inset highlights the LexA binding site of *ydjM*, and the orange inset indicates the start and stop codons of *ydjM*. Yellow and green shading highlight the synonymous and non-synonymous nucleotide substitutions, respectively, within *ydjM*; red shading highlights nucleotide substitutions within the 3’-region of *ydjM*; and blue shading shows nucleotide substitutions within the LexA binding site of *ydjM*.

In the evolutionary phylogeny (Figure 2A), we observed that the seven B2 strains (CFT073, UTI89, 536, APEC O1, S88, ED1 and O127:H6 E2348/69) clustered together according to their pathotypes, and the corresponding bootstrap values were extraordinarily high. Moreover, the five extraintestinal pathogenic *E. coli* strains clustered together, and within the phylogroup B2, they were the most derived subgroup. These seven B2 strains originated from the same progenitor but had different pathotypes. Therefore, we assume that there are certain variations in their DNA regions that may be related to their pathogenicity. To this end, we examined those LCBs that strongly support node 29 of the evolutionary phylogeny but conflict with node 28 (bottom portion of Figure 2A). Strikingly, only one collinear region (1047) was found in our filtered dataset (Table 2), and this region contained one annotated gene named *ydjM*. The product of the gene *ydjM* is an inner membrane protein regulated by LexA and is a member of the SOS network. It has been reported that the SOS response is related to the evolution and dissemination of antibiotic resistance as well as the synthesis and dissemination of virulence [24]. We performed a multiple sequence alignment of the seven DNA segments of the LCB 1047 using the CLUSTALW program [25] with the default settings. We found that almost all of the nucleotide substitutions within the genes were synonymous (Figure 3). However, within the 5’-region of the gene, we found one nucleotide substitution (T to C). This mutation may be closely related to the pathogenicity of the pathogenic strains because the substitution occurs within the LexA binding sites [26]; these results indicate that it is worth performing an experimental evaluation in the future.

**Table 2:** The values of the partitioned branch support (PBS) of Node28 and Node29

**LCB ID**	**PBS**	
	**Node28**	**Node29**
527	12	27
1047^a^	−1	4
435	2	4
428	1	2
539	4	2
350	−7	0
918	−5	0
2325	−3	0
2524	−1	0
1182	−1	0
896	−1	0
3530	0	0
3282	0	0
964	0	0
1231	0	0
991	0	0
1178	0	0
2789	0	0
1781	0	0
1792	0	0
1219	1	0
798	2	0
474	4	0
879	5	0
640	21	0
629	21	0
1225	−5	−1
678	2	−1
974	2	−1
261	3	−1
810	4	−1
692	−7	−3
41	30	−5
366	1	−10
2836^b^	-	-

The 35 LCBs are sorted by their PBS values for node 29 in descending order, then for node 28 in ascending order.

^a^This is the LCB that we are interested in. It supports node 29 while conflicts with node 28.

^b^The seven DNA segments of the LCB 2836 are exactly the same, therefore no value exists.

In the similarity phylogeny (Figure 2B), we observed that three EHEC E subgroups and three EHEC B1 subgroups clustered together, and the corresponding bootstrap value was 100%. These six EHEC strains originated from different progenitors and belong to the same pathotype; therefore, we assume that they may have obtained specific regions related to their pathogenicity through HGT. To test this hypothesis, we examined the LCBs that are only present in these six strains and found that 37 collinear regions satisfied this criterion. Of these LCBs, only 17 have gene annotations. Although most of the gene products are annotated as ‘hypothetical’ or ‘putative’, two genes of phage origin and three type III secretion system (T3SS) effector genes (i.e., *espL2nleB1* and *nleE*) were also found (Additional file 2: Table S2). Nadler et al. [27] found that NleE and NleB play important roles in the interplay between host and pathogen. NleE is sufficient to inhibit NF-kB signaling, which leads to the eradication of the pathogen. In addition, NleB can enhance NleE activity. We performed a BLAST [28] search of the LCBs that contain these three genes against the other 30 genomes under study. Except for strain E2348/69, which belongs to the EPEC B2 phylogroup, no similar genes were found within the genomes of the other 29 strains, including the two outgroups. Therefore, we speculate that LCB 938, which contains the three aforementioned effector protein-coding genes, may be associated with the common pathotypes of these six strains. In addition, we believe that the other 36 collinear regions may also potentially be associated with pathogenicity. Specifically, the LCB 938 could be used as a proxy for the detection of EHEC/EPEC *E. coli* strains.

## Discussion

With the advent of high-throughput DNA sequencing technologies, an increasing number of complete genomes and microbial genomes in particular, have been sequenced in the past ten years. The development of methods for the efficient use of these valuable data in the study of evolutionary biology presents a challenge to biologists. Within each whole genome, there are many hidden features that depict the evolutionary history of the species. These features provide us with a window to the evolutionary history of life on our planet through which comprehensive phylogenetic relationships can be reconstructed [6]. In addition to the two aforementioned major categories of reconstruction methods, other evolutionary markers within complete genomes have also been used. For example, various types of rare genomic changes, such as insertions and deletions (indels), intron positions and overlapping genes, have been used to address specific phylogenetic questions [6,29]. Recently, an approach based on metabolic pathway reaction content was proposed [15]. This method considers the effect of the metabolic networks on phenotypic behavior. In our present work, we used information regarding collinear genomic regions to assess two types of relationships, i.e., evolutionary and phenetic, among 34 *E. coli* strains. Our results suggest that LCBs are suitable for the phylogenomic analysis of the evolutionary relationships among closely related genomes. More interestingly, LCBs could, to a certain extent, reflect the phenetic relationships of the genomes in which they reside.

**Figure 4:**
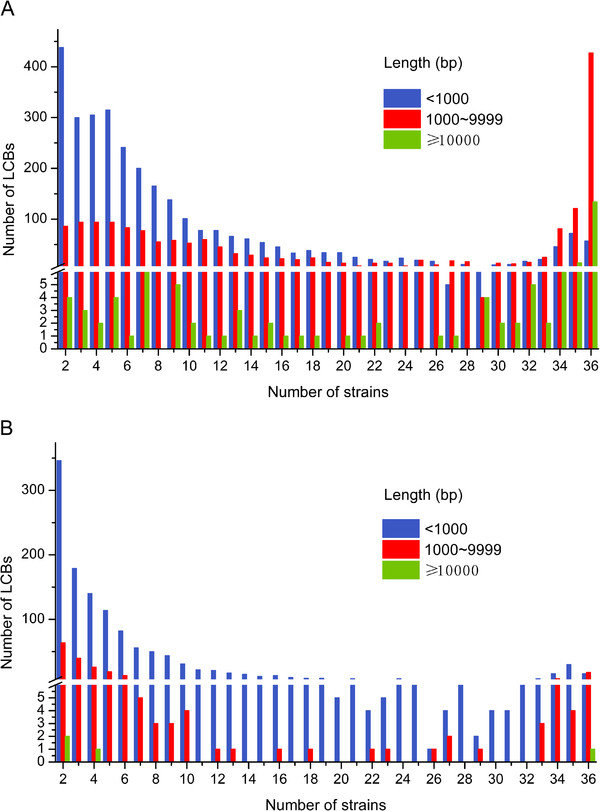
**The number and length distributions of the LCBs.** (**A**) Distributions of the unfiltered LCBs. (**B**) Distributions of the filtered LCBs with a cutoff value of 1.01.

To date, several tools for multiple alignments of whole genomes at the DNA scale have been proposed [11,12]. In this work, we identified a total of 5097 LCBs using the Mugsy program. Figure 4 shows the number and length distributions of LCBs shared by different numbers of strains. Curiously, before being filtered, the number of longer LCBs (>1 kbp) shared by the majority of strains (> = 34 genomes) was greater than that shared by the minority of strains (<= 4 genomes), with the opposite trend observed for the shorter LCBs (Figure 4A). Intuitively, the LCBs shared by the minority of strains should be longer than those shared by the majority of strains. Further examination revealed the existence of many gaps in several LCBs, especially in the longer LCBs. Because even small genomic variations may occasionally change the functions of genes, we filtered the original 5097 LCBs using a strict cutoff value (1.01), which means the length of gaps in each LCB is smaller than 1% of the length of the non-gap regions. After filtering, only 78% and 6% of the original LCBs shared by 2 and 36 strains, respectively, remained (Figure 1). The number of LCBs shared by two strains decreased from 528 to 412, and the number of LCBs shared by all 36 strains decreased from 618 to 35. Moreover, after being filtered, the number of LCBs shared by the majority of strains was less than that shared by the minority of strains irrespective of the length of each LCB (Figure 4B). This observation suggests that longer LCBs tend to inherit genetic materials from their vertical ancestors rather than by HGT. Based on these data, together with the patterns depicted in Figures 4A and B, we conclude that the longer LCBs should have experienced many insertions or deletions during the evolution of the strains.

Although many methods have been designed for the reconstruction of phylogenetic trees among species, many of them are not suitable for inferring intraspecific phylogenetic relationships. Lukjancenko et al. reported that neither 16S rRNA sequences nor MLST gene sets are suitable for the analysis of inter-strain relationships within a species or between closely related species [5]. However, analyses based on whole-genome signatures appear to work well for inter-strain comparisons and the study of closely related genomes. Touchon et al. [1], Ogura et al. [4] and Lukjancenko et al. [5] found that gene-alignment-based methods could group *E. coli* strains in a meaningful way. Touchon et al. built a phylogeny based on 1,878 core genes. Ogura et al. built two phylogenies based on 345 orthologous CDS and a gene repertoire containing 12,940 CDS. Lukjancenko et al. performed a clustering of *E. coli* and related species based on their variable gene content. In a study based on alignment-free inference, Sims et al. [7] discovered that the feature frequency profile (FFP) method could also provide useful information for comparing the whole-genome sequences of *E. coli*. They constructed two phylogenies using the frequency vectors of oligonucleotides with a length of 24 nt. In general, all previously inferred phylogenies can be separated into two categories: those based on the core genes or features, which are similar to our evolutionary phylogeny, and those based on the variable gene content or other genomic features or metabolic pathways, which are similar to our similarity phylogeny. Our evolutionary phylogeny is, overall, congruent with those constructed using similar methods where B2 is the basal phylogroup. In contrast, the *Shigella* strains in our similarity phylogeny were distinctly distant from the other strains. This pattern is in agreement with several previous studies [5,15], whereas there are certain differences relative to other studies [7]. Regarding the methodology, we believe that the comparison using K-mer frequency vectors is similar to the LCB-based comparison used here. The analysis of *K*-mer features, which are good markers for inferring evolutionary histories of organisms across both large and small evolutionary distances and regardless of the genome size variation, may appear to behave differently from other methods when depicting phenotypic behaviors because *K*-mer features contain insufficient functional information. Nonetheless, LCBs, which tend to be conserved between closely related species, could provide more valuable evolutionary and functional information for the analysis of both evolutionary histories and phenotypic behaviors of closely related species and intraspecific genomes in particular. Conversely, because there is minimal conservation of LCBs at great evolutionary distances, it is difficult to provide sufficient information for phylogenomic analyses of distantly related species [30,31].

## Conclusions

Our results demonstrate that collinear regions are suitable for analyzing both the evolutionary history and ecological adaption of *E. coli*. For closely related genomes, collinear regions (LCBs in this study) at the DNA level are reliable genomic features used for phylogenomic analysis while linking the genomic diversity with phenotypic differences in a meaningful way. Our evolutionary phylogeny based on common collinear regions reveals potential signatures exhibiting both meaningful phylogenetic and phenotypic patterns. A comparison of the evolutionary and similarity phylogenies suggests that *Shigella* experienced a convergent evolution event. This group originated from at least three distinct progenitors and evolved into one phenetically similar group. By inspecting certain interesting branches of the evolutionary and similarity phylogenies, we found that the pathogenicity of a strain may be associated with both the arrival of virulence factors through HGT and the modification of genomes via mutations. More interestingly, specific collinear regions could be used as proxies for the detection of certain subgroups of *E. coli*. Given these findings, future experimental validations are needed to confirm the correlation between collinear regions and the pathogenicity inferred in this study.

## Methods

### Bacterial genomes and gene annotations

The genome sequences, which were previously manually re-annotated by experts, were downloaded from the MicroScope database [32] in June 2011. Thirty-four strains of *E. coli*, including 28 *E. coli* strains and six *Shigella* strains that belong to the *E. coli* species [14], as well as the two outgroups ATCC_35469^T^ and Typhimurium LT2 from *E. fergusonii* and *S. enterica*, respectively, were used in this study (Table 3). This set of strains covers all of the main phylogenetic groups (A, B1, B2, D, E and F) [33] and various pathogenic behaviors (commensal, ExPEc and InPEc) of *E. coli* strains as well as four phylogenetic groups (S1, S3, SD1 and SS) [14] of *Shigella* strains (the pathotype of which are marked as Shigellosis). The phylogenetic groups and pathotypes of these strains were obtained from previous publications [7,15] and the GOLD database [34].

**Table 3:** Main characteristics of the strains used in this study

**Strain**	**Phylogenetic group**	**Pathotype**^**a**^	**Genome size (Mb)**	**Accession number (MicroScope)**
***Escherichia coli***				
K12 W3110	A	Commensal	4.5	GBKW3110_AC_000091
HS	A	Commensal	4.5	EcHS_A NC_009800
ATCC8739	A	Commensal	4.6	EcolC_NC_010468
K12 DH10B	A	Commensal	4.6	ECDH10B_NC_010473
K12 BW2952	A	Commensal	4.5	BWG_NC_012759
BL21(DE3)	A	Commensal	4.5	ECBD_NC_012947
B REL606	A	Commensal	4.5	ECB_NC_012967
K12 MG1655	A	Commensal	4.5	U00096
E24377A	B1	InPEc(ETEC)	4.9	EcE24377A_NC_009801
SE11	B1	Commensal	4.8	ECSE_NC_011415
IAI1	B1	Commensal	4.6	ECIAI1_EIAI1v2
55989	B1	InPEc(EAEC)	5	EC55989_EC55v2
O103:H2 12009	B1	InPEc(EHEC)	5.3	ECO103_NC_013353
O26:H11 11368	B1	InPEc(EHEC)	5.6	ECO26_NC_013361
O111:H- 11128	B1	InPEc(EHEC)	5.2	ECO111_NC_013364
CFT073	B2	ExPEc	5.1	c NC_004431
UTI89	B2	ExPEc	5	UTI89_C NC_007946
536	B2	ExPEc	4.8	ECP_NC_008253
APEC O1	B2	ExPEc	5	APECO1_NC_008563
O127:H6 E2348/69	B2	InPEc(EPEC)	4.9	E2348C_NC_011601
S88	B2	ExPEc	4.9	ECS88_ECOS88V2
ED1a	B2	Commensal	5.1	ECED1_ED1av2
UMN026	D	ExPEc	5.1	ECUMNv2_ESCUMv2
O157:H7 EDL933	E	InPEc(EHEC)	5.4	Z NC_002655
O157:H7 Sakai	E	InPEc(EHEC)	5.4	Ecs NC_002695
O157:H7 EC4115	E	InPEc(EHEC)	5.4	ECH74115_NC_011353
SMS-3-5	F	Commensal	5	EcSMS35_NC_010498
IAI39	F	ExPEc	5	ECIAI39_EIAI39v2
***Shigella***				
*S. boydii* 4 227 (Sb 227)	S(S1)	Shigellosis	4.4	SBO NC_007613
*S. flexneri* 2a 301 (Sf 301)	S(S3)	Shigellosis	4.5	SF NC_004337
*S. flexneri* 2a 2457 T (Sf 2457 T)	S(S3)	Shigellosis	4.5	S NC_004741
*S. flexneri* 5 8401 (Sf 8401)	S(S3)	Shigellosis	4.5	SFV_NC_008258
*S. dysenteriae* 1 197 (Sd 197)	S(SD1)	Shigellosis	4.3	SDY NC_007606
*S. sonnei* 046 (Ss 046)	S(SS)	Shigellosis	4.7	SSO_NC_007384
**Outgroup**				
*E. fergusonii* ATCC 35496	Outgroup	Commensal	4.5	EFER_EFERv2
*S. enterica* enterica LT2	Outgroup	Pathogen	4.7	STM NC_003197

^a^InPEc: Intraintestinal pathogenic *E. coli*, ExPEc: Extraintestinal pathogenic *E. coli*.

ETEC: enterotoxigenic *E. coli*, EAEC: enteroaggregative *E. coli*, EHEC: enterohemorrhagic *E. coli*, EPEC: enteropathogenic *E. coli*.

### Multiple whole-genome alignments of the strain genomes

Whole-genome comparisons were performed using the Mugsy program version 1.2.2, which is computationally efficient and can effectively align closely related whole genomes compared to other tools [12]. All chromosomes from the 36 genomes were aligned using the parameters “-distance = 1000” and “–minlength = 30”, which specify the maximum genomic distance between adjacent anchors and the minimum block length, respectively. To guarantee the quality of the Mugsy output file, a list of LCBs was further filtered using a cutoff value (score ≤ 1.01). This value was defined as follows:

(1) $$score=\frac{L_{c}\times n}{\sum_{i}^{n}l_{i}}$$

Where *L*_*c*_ is the length of the LCB (including the gap), *n* is the number of strains in which this LCB is present and *l*_*i*_ is the length of the LCB from strain *i* (excluding gaps). When an LCB was contained within another LCB, the internal LCB was discarded and the longer LCB was retained. This set of filtered LCBs was then used to perform the phylogenomic analysis.

### Phylogenies based on LCBs

Two tree-construction methods were used: (*i*) an evolutionary phylogeny based on core LCBs that are shared by all the studied genomes and (*ii*) a similarity phylogeny based on variable LCBs that are absent in at least one genome.

#### Evolutionary phylogeny

The LCBs shared by all of the studied genomes were assembled into a concatenated LCB matrix in NEXUS format using the ASAP program [35]. A maximum parsimony phylogenomic tree was then constructed using PAUP* 4b10 [36] using the heuristic search option with 200 random taxon additions and TBR branch swapping. The partitioned branch supports (PBSs) [37], which identify the relative contribution of each of the data partitions to the concatenated tree at each node, were also calculated using ASAP. Positive, negative and zero PBS values signify character support, conflict and neither support nor conflict, respectively.

#### Similarity phylogeny

The similarity phylogeny was constructed by the neighbor joining methods using the PHYLIP package version 3.68 [38]. Briefly, a matrix of LCBs was constructed with values of 1 or 0 where each row corresponds to an LCB and each column represents a genome. A cell (*i,j*) in the matrix is equal to 1 if LCB *i* is present in genome *j* or equal to 0 if the LCB is absent. The Jaccard distances, defined below, were calculated and used to generate the distance matrix for the phylogenetic inference. The LCBs present in only one genome or in all of the genomes were not included in this analysis. Bootstrap values were computed for each inner node by re-sampling the rows of the matrix 1000 times.

(2) $$J_{A,B}=\frac{M_{10}+M_{01}}{M_{10}+M_{01}+M_{11}}$$

In the above equation, *M*_10_ represents the number of LCBs that are present in genome *A* but absent from genome *B*. *M*_01_ represents the number of LCBs that are absent from genome *A* but present in genome *B*. Finally, *M*_11_ represents the number of LCBs that are present in both genomes *A* and *B*.

## Abbreviations

MLST: Multilocus sequence typing; LCB: Locally collinear block; HGT: Horizontal gene transfer; VF: Virulence factor; T3SS: Type III secretion system; FFP: Feature frequency profile; PBS: Partitioned branch support; InPEc: Intraintestinal pathogenic *Escherichia coli*; ExPEc: Extraintestinal pathogenic *Escherichia coli*; ETEC: Enterotoxigenic *Escherichia coli*; EAEC: Enteroaggregative *Escherichia coli*; EHEC: Enterohemorrhagic *Escherichia coli*; EPEC: Enteropathogenic *Escherichia coli*.

## Competing interests

The authors declare that they have no competing interests.

## Authors’ contributions

KL designed the work and helped to draft the manuscript. YZ performed the work and wrote the manuscript. All authors read and approved the final manuscript.

## Supplementary Material

Additional file 1 **Table S1.** List of genes along the LCBs which are specific to six *Shigella* strains. Click here for file

Additional file 2 **Table S2.** List of genes within the LCBs that are specific to six enterohemorrhagic *E. coli*. Click here for file
